# ERRATUM: Perception of the nursing team of a surgical center regarding hospital accreditation at a university hospital

**DOI:** 10.1590/1980-220X-REEUSP-2024-ER04en

**Published:** 2024-09-30

In the article “Perception of the nursing team of a Surgical Center regarding Hospital Accreditation at a University Hospital”, with the DOI: https://doi.org/10.1590/S0080-623420150000700004, published by the journal: “Revista da Escola de Enfermagem da USP, volume 49 (spe) of 2015, p. 21–27, on page 27:

**Now read:**

## Financial Support

This study was financed in part by the Coordenação de Aperfeiçoamento de Pessoal de Nível Superior – Brasil (CAPES) – Finance Code 001.

