# Supplementary material for: ERRATUM: Perception of the nursing team of a surgical center regarding hospital accreditation at a university hospital
Source: Rev Esc Enferm USP. 2024 Sep 30;58:e2024ER04. doi: 10.1590/1980-220X-REEUSP-2024-ER04en (PMC11524264; doi:10.1590/1980-220X-REEUSP-2024-ER04en)
Supplement: Translation

# Translation: ERRATA: Percepção da equipe de enfermagem do centro cirúrgico acerca da acreditação hospitalar em um hospital universitário

No artigo “Percepção da equipe de enfermagem do Centro Cirúrgico acerca da Acreditação Hospitalar em um Hospital Universitário”, com número de DOI: https://doi.org/10.1590/S0080-623420150000700004, publicado na Revista da Escola de Enfermagem da USP, volume 49 (Esp) de 2015, p. 21–27, na página 27:

**Leia-se:**

## Apoio financeiro

O presente trabalho foi realizado com apoio da Coordenação e Aperfeiçoamento de Pessoal de Nível Superior – Brasil (CAPES) – Código de Financiamento 001.
